# Top-Down Inputs Enhance Orientation Selectivity in Neurons of the Primary Visual Cortex during Perceptual Learning

**DOI:** 10.1371/journal.pcbi.1003770

**Published:** 2014-08-14

**Authors:** Samat Moldakarimov, Maxim Bazhenov, Terrence J. Sejnowski

**Affiliations:** 1 Howard Hughes Medical Institute, The Salk Institute for Biological Studies, La Jolla, California, United States of America; 2 The Institute for Neural Computation, University of California, San Diego, La Jolla, California, United States of America; 3 Department of Cell Biology and Neuroscience, University of California, Riverside, Riverside, California, United States of America; 4 Division of Biological Sciences, University of California, San Diego, La Jolla, California, United States of America; Philipps-University Marburg, Germany

## Abstract

Perceptual learning has been used to probe the mechanisms of cortical plasticity in the adult brain. Feedback projections are ubiquitous in the cortex, but little is known about their role in cortical plasticity. Here we explore the hypothesis that learning visual orientation discrimination involves learning-dependent plasticity of top-down feedback inputs from higher cortical areas, serving a different function from plasticity due to changes in recurrent connections within a cortical area. In a Hodgkin-Huxley-based spiking neural network model of visual cortex, we show that modulation of feedback inputs to V1 from higher cortical areas results in shunting inhibition in V1 neurons, which changes the response properties of V1 neurons. The orientation selectivity of V1 neurons is enhanced without changing orientation preference, preserving the topographic organizations in V1. These results provide new insights to the mechanisms of plasticity in the adult brain, reconciling apparently inconsistent experiments and providing a new hypothesis for a functional role of the feedback connections.

## Author Summary

The specificity of visual perceptual learning suggests the primary visual cortex, area V1, is the site of neural plasticity that underlies learning. However, V1 does not exhibit topographic changes observed in other sensory cortices with learning, and changes observed in V1 following perceptual learning are contradictory. In a spiking neuronal model of cortical network, we show that feedback inputs to V1 can change the response properties of V1 neurons without changing the topographic organization in V1. The feedback model reconciles previous experiments and provides a new hypothesis for a functional role of the feedback connections.

## Introduction

The adult brain remains plastic long after the developmental period [1]. This ability to remain plastic is fundamental for the adult brain to be able to learn and adapt in the ever-changing sensory environment. However, we do not fully understand the limits and mechanisms of the adult brain plasticity [2]. Perceptual learning – improvement in perception due to experience with stimuli – has been used to explore plasticity in sensory cortices [3]. In particular, stimulus specificity of the improvements of orientation discrimination [4]–[6] suggests that the primary visual cortex, area V1, where neurons have small receptive fields, is the site for cortical changes that underlie the improvement of orientation discrimination. However, it is still unclear what cellular and synaptic changes in V1 are responsible for such perceptual learning.

**Figure 1:**
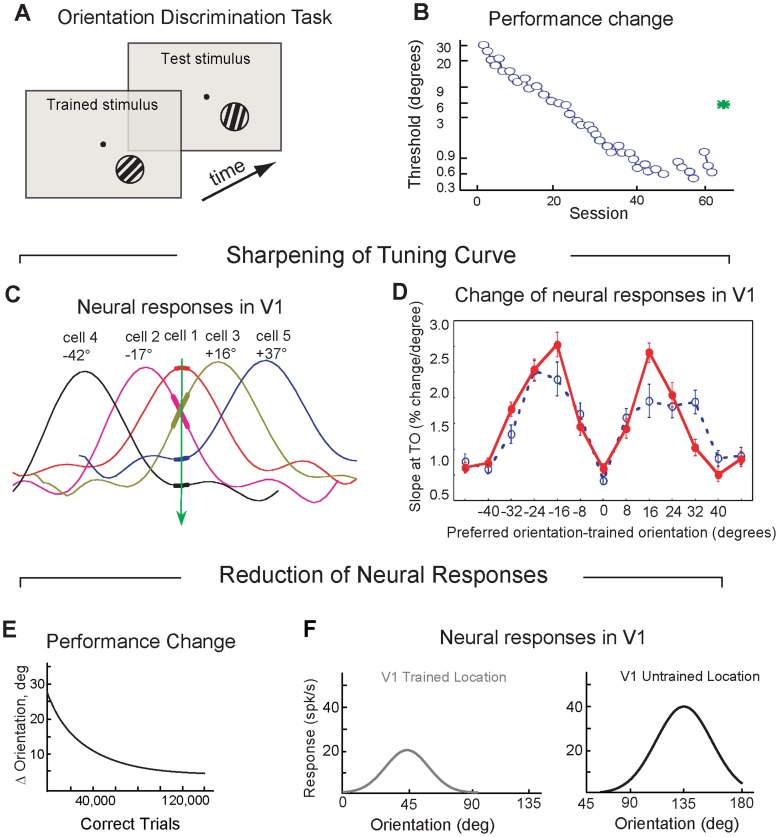
Effect of training on orientation discrimination in V1 neurons. **A.** Orientation discrimination task. Subjects reported whether the test orientation was tilted clockwise or anticlockwise with respect to the trained orientation (from [7]). **B.** Performance in orientation discrimination task (from [7]). **C.** Orientation tuning curves of five sample V1 neurons (from [7]). **D.** Slope measured at the trained orientation for trained neurons (solid red line) and naïve neurons (dashed blue line) (from [7]). **E.** Performance in orientation discrimination task in another experiment (from [8]). **F.** Neural responses in V1 in the trained location (grey line) and an untrained location (black) (from [8]).

Reports of learning-dependent changes of V1 neurons are inconsistent: Learning to discriminate orientations of visual stimuli in one study resulted in sharpening orientation tuning curves in a subgroup of V1 neurons [7] (Fig. 1A–D); others found instead that responses to the trained orientation reduced in V1 neurons [8] (Fig. 1E,F). In both studies, V1 neurons responded preferably to the same orientations as they did before learning, thus preserving the topographic organization in V1.

Previous models of visual perceptual learning based on plasticity of the recurrent and feedforward connections [9]–[10] could neither explain the observed stability of orientation preferences of V1 neurons nor reconcile the findings of the aforementioned experiments.

**Figure 2:**
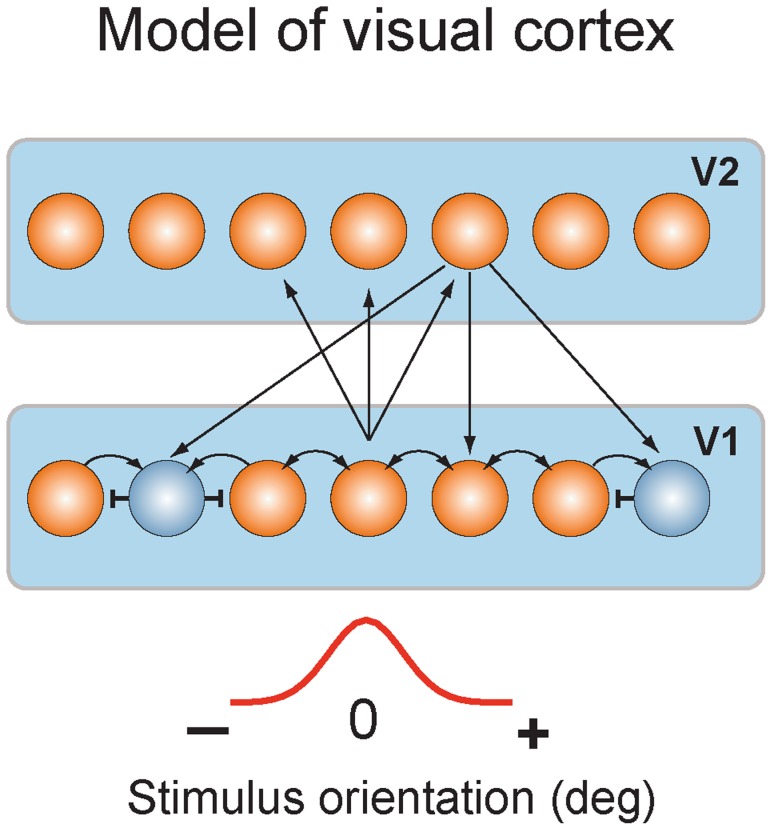
Model of visual cortex. Layer one represented layer 2/3 of V1 and contained excitatory and inhibitory neurons. Layer two represented V2 area and contained excitatory neurons. V1 neurons were connected through recurrent (excitatory and inhibitory) connections. V1 neurons received bell-shaped input, which mimicked orientation-tuned input from layer 4 neurons of V1. V1 excitatory neurons sent convergent feedforward projections to excitatory V2 neurons. V2 neurons projected back to all V1 neurons. Stimuli of different orientations were modeled by shifting the center of the input along one-dimensional network.

Here we suggest that learning-dependent changes in V1 cortex involve top-down projections into V1 from higher cortical areas. In a spiking neural network model of visual cortex (Fig. 2), we showed that repeated stimulus presentations resulted in strengthening the feedforward connections from V1 neurons to neurons in a higher visual area (such as area V2). Greater activity in V2 in turn led to strengthening the feedback connections from V2 to V1 neurons, which helped to maintain the stability of the V1–V2 network through shunting inhibition [11]–[12]. As a consequence of the shunting inhibition, V1 neurons had enhanced orientation selectivity and responded to a narrower range of orientations. The diffuse nature of the feedback inputs to V1 neurons allowed improvement of orientation selectivity in V1 neurons without changing their orientation preferences, thus preserving the map of orientation representations in V1.

The feedback model reproduced both the previously observed sharpening and reduction of neural responses, but not in the same V1 neurons. We assume that previous studies might have recorded from different populations of V1 neurons. We suggest that the stability of cortical representations in V1 may be critical for normal visual processing, and learning-dependent plasticity of visual cortex could be based on mechanisms that preserve the topography of cortical maps.

## Results

### Shunting inhibition of V1 neurons

**Figure 3:**
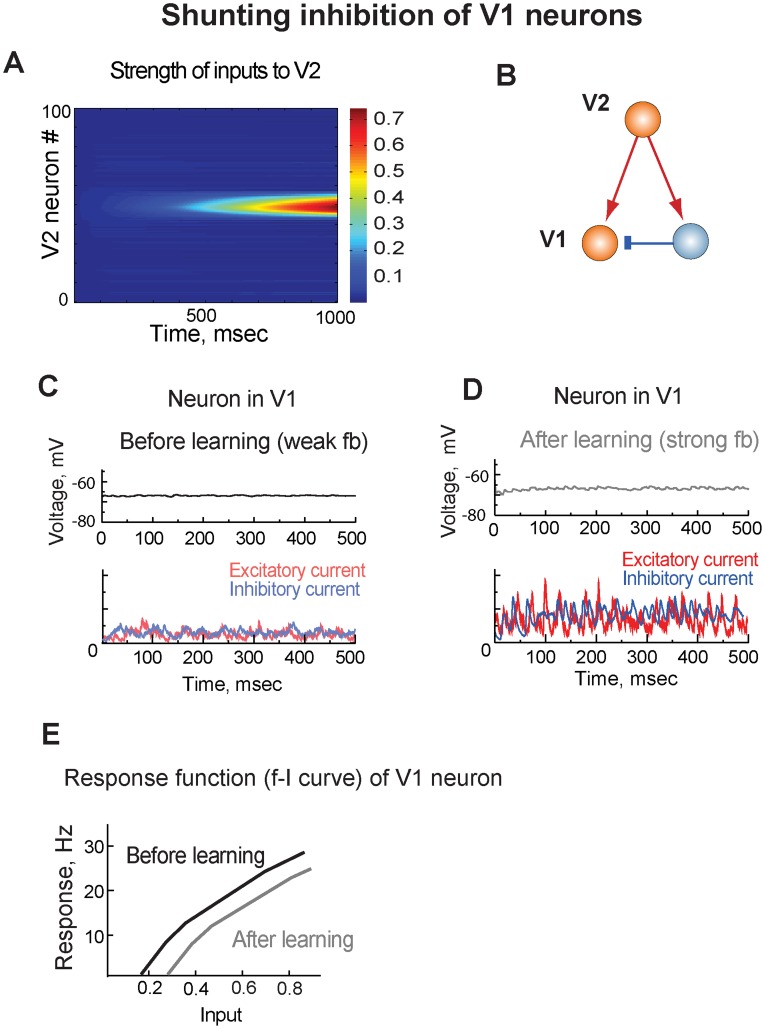
Shunting inhibition of V1. **A.** With learning, feedforward connections from a population of V1 neurons to a population V2 neurons strengthened. **B.** The excitatory V2 neurons projected to both the excitatory and inhibitory neurons in V1. **C.** ***Top***. Membrane potential of a V1 neuron before training. ***Bottom***. Excitatory and inhibitory input currents to the V1 neuron before training. **D.** ***Top***. Membrane potential of the V1 neuron after training. ***Bottom***. Excitatory and inhibitory currents to the V1 neuron after training. **E.** Response function (f-I curve) of a V1 neuron. The neuron did not respond to the trained stimulus, and the response function was tested by injecting current to the neuron.

To explore mechanisms of learning-induced changes in V1 we designed a simple network model consisting of two cortical areas (V1 and V2) and including plastic feedforward and feedback connections (Fig. 2; see also Models). In the model, a stimulus was presented to V1 neurons on many repetitions, which resulted in strengthening the feedforward connections from V1 to V2 neurons (Fig. 3A). Higher levels of activity in V2 neurons in turn led to strengthening the feedback connections from V2 back to V1 neurons. The excitatory feedbacks targeted both the excitatory and inhibitory neurons in V1 (Fig. 3B) and resulted in balanced recurrent excitatory and inhibitory currents in V1, which canceled each other at the resting potential of V1 neurons leading to shunting inhibition of V1 neurons (Fig. 3C). Strengthening the feedback inputs with learning did not change the balance but increased the amplitudes of both the recurrent excitatory and inhibitory currents (Fig. 3D). Thus, although the feedback inputs were excitatory, they suppressed activity in V1 neurons through shunting inhibition mechanism, thus maintaining stability of the network.

Due to the diffuse nature of the feedback inputs in the model, the shunting inhibition affected responses of all V1 neurons. By applying a range of input stimuli, we tested f-I curve in a V1 neuron that did not respond to the trained stimulus but received the modified feedback input. After training, the f-I curve of the V1 neuron shifted to the right, resulting in a subtractive effect on neural responses (Fig. 3E). This was because the strong feedback inputs resulted in stronger recurrent excitatory and inhibitory input currents, which decreased input resistance at the resting potential of the neuron. As we the depolarized membrane potential by applying a direct current to the V1 neuron, the driving force of the inhibitory current increased and the driving force of the excitatory current to the neuron decreased causing shunting inhibition in the neuron. Thus, shunting inhibition can reduce neural responses by counteracting the driving stimulus, as was previously predicted [12].

### Different enhancement of orientation selectivity in V1 neurons after learning

**Figure 4:**
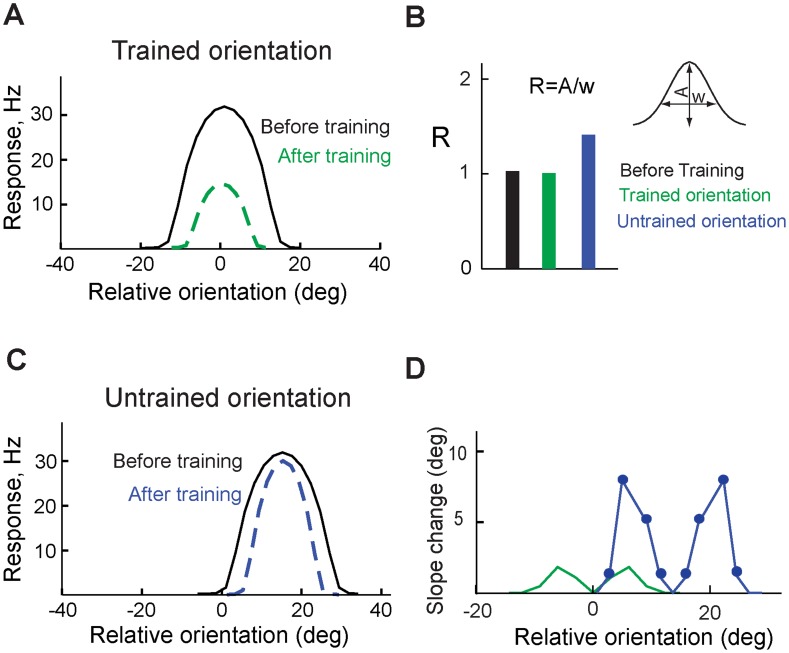
Enhancement of stimulus selectivity. **A.** Tuning curve of a V1 neuron that preferred the trained orientation; before (black) and after (green) training. **B.** Amplitude-to-width ratio (R) of the tuning curve before (black) and after training (green) in a neuron that preferred the trained orientation, and in a neuron that preferred different orientation (blue). **C.** Tuning curve of a V1 neuron that preferred orientation different than the trained one; before (black) and after (blue) training. **D.** Slope change of the tuning curve of the V1 neuron the preferred the trained orientation (green) and another V1 neuron that preferred another orientation (blue line with filled circles).

After training the model with a stimulus, we observed that both amplitude and width of tuning curve of a V1 neuron that preferred the trained orientation reduced due to the shunting inhibition (Fig. 4A,B). However, slope of the neuron's tuning curve did not change significantly (Fig. 4D). We refer to this change in the tuning curve as “reduction” of the response. In another V1 neuron, for which the trained orientation was not the preferred one, reduction of the width of the tuning curve was greater than reduction of the response amplitude (Fig. 4B,C), which steepened the slope of the tuning curve (Fig. 4D). We refer to this change as “sharpening” the tuning curve.

Thus, the reduction of responses was observed in V1 neurons that preferred the trained orientation, and sharpening of tuning curves was observed in V1 neurons that responded to the trained orientation but preferred orientations different from the trained one.

### Stimulus specificity of tuning curve changes

**Figure 5:**
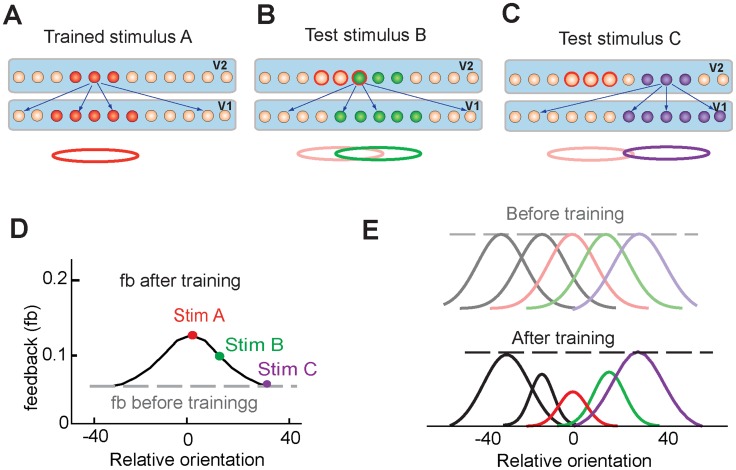
Stimulus specificity of tuning curve changes. **A.** Training the model with stimulus ***A*** strengthened the feedforward and feedback connections between stimulus-specific populations of V1 and V2 neurons. **B.** A test stimulus ***B*** (green) activated population of neurons in V1 and V2 that included some neurons with the modified synapses. The red circle shows where the trained stimulus ***A*** was presented to the model. **C.** Another test stimulus ***C*** (purple) activated populations of V1 and V2 neurons that did not include neurons with modified synapses. **D.** Before training, the strength of the feedback inputs was equal for any applied stimulus (grey dashed line). After training, the strength of feedback inputs depended on the applied stimulus (black solid line). **E.** Stimulus specificity of changes to tuning curves. Peaks of tuning curves did not change, but amplitudes and widths of the tuning curves changed depending on the distance from the trained orientation.

To understand causes of different changes of the tuning curves of V1 neurons (Fig. 4), we explored stimulus dependence of neural changes in V2. Learning resulted in strengthening connections from excitatory neurons representing the trained stimulus in V1 to excitatory neurons representing the trained stimulus in V2 (Fig. 5A). Higher activity in those V2 neurons resulted in strong feedback inputs to all V1 neurons (Fig. 5D), which led to overall reduction of neural responses in V1 due to shunting inhibition (Fig. 5E, red lines). A novel stimulus activated a population of V2 neurons that included some V2 neurons with previously modified connections and other V2 neurons with unmodified connections (Fig. 5B). Hence, the feedback inputs from the experienced V2 neurons were combined with feedback inputs from naïve V2 neurons, which resulted in a lesser increase of the overall feedback inputs to V1 neurons (Fig. 5D) and led to a sharper tuning curve (Fig. 5E, green lines). Finally, a stimulus that activated V2 neurons with unmodified synapses (Fig. 3C) did not change the feedback inputs to V1 (Fig. 5D), therefore, no changes of tuning curves were observed in V1 neurons (Fig. 5E, purple lines). Thus, after learning, strength of the feedback input depended on orientation of a stimulus: The feedback inputs were the strongest for the trained orientation and weakened as stimulus orientation moved away from the trained orientation.

### Effect of feedbacks on response functions in V1

To understand how varying strength of the feedback inputs led to either firing rate reduction or sharpening of the tuning curves of V1 neurons (Fig. 4A and 4C), we analyzed how recurrent interactions within V1 network change response properties of V1 neurons by comparing the responses of two V1 neurons positioned at different locations with respect to the stimuli.

**Figure 6:**
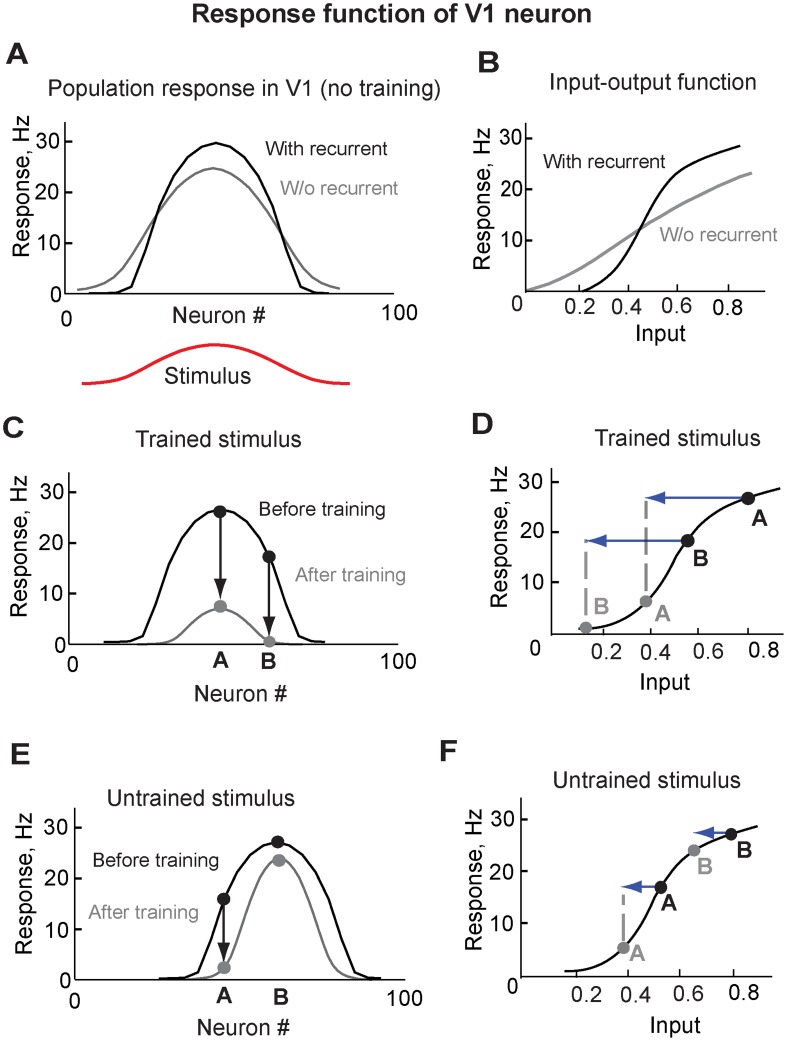
Effect of feedbacks on population response in V1. **A.** Population response in V1 to a stimulus before training, with (black) and without (grey) recurrent connections among V1 neurons. **B.** Input-output function in V1 neurons, with (black) and without (grey) recurrent connections among V1 neurons. **C.** Population response in V1 to the trained stimulus (preferred stimulus for neuron ***A***) before and after training. **D.** Responses of neurons ***A*** and ***B*** to the trained stimulus (preferred for neuron ***A***) changed due to feedback. Black line indicates input-output relation in V1. Blue arrows indicate the strength of feedback inputs. Black and grey filled circles represent neural response of neurons ***A*** and ***B*** to the trained stimulus before and after training. **E.** Population response in V1 to a novel stimulus (preferred for neuron ***B***), before and after training with the stimulus preferred for neuron ***A***. **F.** Responses of neurons ***A*** and ***B*** to the novel stimulus (preferred for neuron ***B***) changed due to feedback. Black line indicates input-output relation in V1. Blue arrows indicate feedback inputs. Black and grey filled circles represent neural responses to stimulus preferred for neuron ***B*** before and after training.

In agreement with experimental data from V1 [13]–[15], recurrent interactions among V1 neurons increased responses of strongly responding V1 neurons and suppressed responses of weakly responding V1 neurons (Fig. 6A). This occurred because recurrent interactions effectively increased nonlinearity of input-output relations in V1 neurons (Fig. 6B). The new input-output function had an intermediate area where the slope changed most strongly. We refer to this region as an area of strong nonlinearity.

The trained stimulus, which was the preferred stimulus for neuron **A** (Fig. 6C), evoked strong feedback inputs and resulted in proportional reduction of neural responses in both strongly (e.g. neuron **A**) and weakly (e.g. neuron **B**) responding V1 neurons (Fig. 6C). This proportional reduction was because the strong feedback equally affected neurons **A** and **B** by shifting responses in both neurons beyond the area of strong nonlinearity (Fig. 6D).

A novel stimulus, which was the preferred for neuron **B**, evoked weaker feedback inputs and led to the sharpening of V1 neural responses (Fig. 6E). This sharpening was because not-so-strong feedback affected neurons **A** and **B** differently. The feedback shifted the response of neuron **A** beyond the area of strong nonlinearity, which led to a strong reduction of its response. In contrast, the response of neuron **B** remained outside the area of strong nonlinearity and displayed only a minor decrease (Fig. 6F). This difference in the firing rate change of different V1 neurons resulted in sharpening of the population response (Fig. 6E).

Thus, the strong nonlinearity of the input-output function of V1 neurons and the varying strength of the feedback inputs produced different changes in the neural population, reducing the responses of some neurons and sharpening the tuning curves of others. These changes of the population responses observed in our model were similar to the reported changes of the tuning curves in V1 neurons following perceptual learning [7]–[8].

## Discussion

In the primary auditory, somatosensory, and motor cortices, learning leads to dramatic increases in the number of neurons that represent the trained stimuli [16]–[20]. In the primary visual cortex (V1), topographic reorganization was observed following severe sensory changes [21]–[23], which raised the question of whether such changes in V1 can also occur during normal perceptual learning [24]. Topographic reorganization in V1 have not been reported in any study following perceptual learning. Here we have shown cellular and synaptic mechanisms of cortical plasticity that can preserve the topographic organization in area V1 while allowing changes to the properties of V1 neurons. These results suggest that learning to discriminate simple visual stimuli may evoke changes that are different from the topographic reorganization observed in other sensory cortices. The feedback mechanism for learning proposed here may not, however, be limited to the visual system. Other sensory modalities may share similar mechanisms, depending on the particular learning task.

### Scope of the model

Our model pertains to experiments that involve learning to discriminate low-level features of visual stimuli presented at a particular location within receptive field of V1 neurons. We explored here if learning-induced changes in V1 were not due to synaptic plasticity of short-range recurrent connections among nearby V1 neurons within a hypercolumn but instead were due to changes in feedback inputs to V1 neurons from neurons in higher visual areas. There may be other types of plasticity in the long-range horizontal connections between distant hypercolumns that can explain other types of learning experiments involving complex tasks and large stimuli [25]–[27].

### Robustness of the model

Reduction of neural responses and sharpening of tuning curves in our model resulted from strong nonlinearity of input-output relations in V1 neurons and varying strengths of stimulus evoked feedback inputs to V1 neurons. The intrinsic nonlinearity of input-output relations in V1 neurons was further amplified by recurrent interactions. The variation of strengths of the feedback inputs resulted from the assumption that a population of V2 neurons representing a stimulus had a finite size. Therefore, the trained and novel stimuli evoked responses in an overlapping but different neuronal populations, which resulted in different feedback inputs to V1 neurons upon presentation of the trained vs. novel stimuli. Thus, both reduction of the neural responses and sharpening were observed in V1 network but in different populations of V1 neurons with respect to the location of stimulation. The learning process in the model was incremental: More training resulted in stronger feedforward and feedback connections, and therefore led to stronger reduction and sharpening. Robustness of the results was tested by varying the synaptic weights in the model, which produced consistent behavior over a range of parameter values. Other aspects of the model were based on neural data and did not affect the conclusions.

### Shunting inhibition and gain modulation

In a previous study, driving a spiking neuron with balanced excitation and inhibition currents changed the slope of the gain function (input-output function) [11]. The changes of the gain functions were caused by shunting inhibition due to balanced excitation and inhibition and noise in the input currents. However, in perceptual learning experiments analysis of noise in V1 neural responses before and after learning did not show any change in the noise level [7]. In the current model, input noise did not change with training because the firing rates in V1 did not increase with learning, and firing rates in V2 were maintained through the feedback mechanism. Thus, in the model, balanced excitatory and inhibitory feedback currents were solely responsible for the subtractive inhibition in V1 neurons, in agreement with the experiments.

### Learning and adaptation

In our model, the feedback mechanism of learning resulted in symmetric changes of the tuning curves in V1. However, tuning curves of V1 neurons can in fact adapt and become asymmetric in response to repeated stimulus presentation [28]. This adaptation may be due to short-term synaptic depression and facilitation in the recurrent connections, which can result in asymmetric changes of the tuning curves. In contrast, the changes in V1 discussed in our study are due to long-term changes in the feedforward and feedback connections.

Both adaptation and learning could take place during stimulation and two processes could interact with each other [29]–[31]. The critical distinction between adaptation and learning is that learning, unlike adaptation, is task dependent. Both adaptation and learning can take various forms due to diversity of plasticity mechanisms (Hebbian, homeostatic, reinforcement, etc) that could be evoked by the stimulus [32]. However, with learning to perform a task, changes that enhance performance are reinforced, and changes that do not improve performance are suppressed, a distinction that may depend on a reinforcement signal. We have not included a reinforcement signal in our model, and instead assumed that plasticity of feedforward and feedback connections somehow depended on the task. Although recurrent connections did not contribute to learning in our model, they could be the basis for adaptation. Thus, both learning and adaptation can occur simultaneously and their relative contribution can be context specific.

### Specificity of perceptual learning

In many experiments on visual perceptual learning, the improvements of perception were stimulus specific [4]–[6], suggesting that early visual areas were possible sites for the changes underlying visual perceptual learning. However, under some conditions learning can be generalized to other locations in the visual field [29], [33]. These observations can be explained by stimulus-invariant changes occurring in higher decision-making cortical areas that augment those in lower visual areas.

In our model of learning-induced plasticity of V1, we did not consider if/how changes in V1 could improve perception. In order to understand how perceptual learning could be generalized to other stimuli and locations, we would need to expand the model to include read-out decision-making circuits. However, modeling plasticity in the higher cortical areas, in addition to the changes in V1, is beyond the scope of this study, which is focused on changes that have been observed in V1.

### Conclusion

Our study suggests that two previously reported inconsistent experimental results [7]–[8] may represent recordings from two different populations of V1 neurons: One population of neurons that prefer the trained stimulus, and another that prefer different stimuli but still respond to the trained stimulus.

Our model also predicts that learning results in strengthening feedback inputs into V1, which cause shunting inhibition in V1 neurons. This novel mechanism of learning can be tested by measuring input resistances in relevant V1 pyramidal cells during in vivo stimulation before and after learning.

Despite of limited nature of learning-induced changes in V1, these changes could contribute to other learning-dependent changes in visual cortex, because of the hierarchical organization of the visual system [34]–[35]. Indeed, a learning-induced sharpening of orientation tuning curves was observed in an extrastriate visual area V4 [36], which could be at least partially due to more sharply-tuned inputs from V1.

Feedback projections are ubiquitous in cortex [37]–[38], but despite recent progress [39]–[42], little is known about functional roles of feedback connections in cortical plasticity. Our work presents a new hypothesis for the role of the feedback connections in cortical plasticity. We propose that top-down inputs are plastic and involved in perceptual learning. This prediction can be tested by blocking synaptic plasticity in extrastriate visual cortical areas and observing whether this would diminish learning-induced improvement of behavior.

## Models

### Spiking neuron network model of visual cortex

We explore the feedback hypothesis in a spiking neural network model of the visual cortex (Fig. 2). The model consisted of two layers. Layer one represented the primary visual cortex V1 and layer two represented a higher cortical area V2.

In the biological visual cortex, feedforward inputs from V1 drive activity in V2 neurons, but feedback inputs from V2 neurons back to V1 do not drive but modulate activity in V1 [43]–[44], thus avoiding a strong positive feedback loop that could lead to uncontrolled epileptic-like oscillations [45]. In the model, V1 neurons activated V2 neurons through the convergent feedforward connections (Fig. 2). The diffuse feedback inputs from V2 did not drive V1 neurons, but altered their response functions through shunting inhibition mechanism [11]–[12]. Shunting inhibition in the model was due to the balanced excitatory and inhibitory to the excitatory V1 neurons, which modulated neural responses in V1 neurons by subtracting neural responses to driving stimuli.

#### V1 layer

In the primary visual cortex, orientation selectivity of layer 2/3 neurons are formed by orientation-tuned feedforward inputs from layer 4 neurons, and by recurrent interactions among layer 2/3 neurons [15]. Orientation selectivity of layer 4 simple cells in turn is formed by feedforward inputs from arranged LGN cells [46]. In this model, we did not model LGN and layer 4 neurons. Layer 2/3 neurons receive a bell-shaped input, which represented orientation tuned inputs from layer 4 cells. The neurons in V1 layer were organized with preferred orientations evenly spaced along one-dimensional network. Stimuli of different orientations were modeled by shifting the center of the input along one-dimensional V1 network.

V1 layer contained 100 excitatory and 25 inhibitory neurons, which represented layer 2/3 of V1. Both the excitatory and inhibitory neurons were simulated using Hodgkin-Huxley type neuron model with sets of parameters resulting in regular spiking activity [47].

#### Excitatory neurons



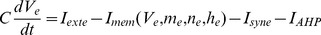
(1)

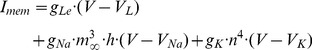
(2)

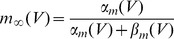
(3)

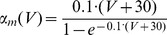
(4)

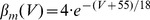
(5)

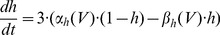
(6)

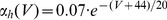
(7)

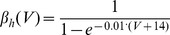
(8)

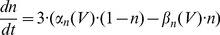
(9)

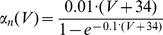
(10)

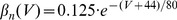
(11)

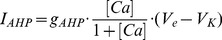
(12)

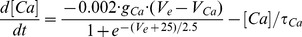
(13)where *V_e_* is membrane potential of an excitatory neuron, *m*, *h*, *n* are the gating variables of voltage-gated sodium and potassium channels, *g_L_* is maximum conductance of the leak current, *g_Na_* is maximum conductance of sodium current, *g_K_* is maximum conductance of potassium current, *V_L_* is reversal potential of the leak current, *V_Na_* is reversal potential for sodium currents, *V_K_* is reversal potential for potassium currents, *[Ca]* is the intracellular concentration of free calcium ions, and *g_AHP_* is the maximum conductance of the calcium-dependent potassium current.

Model parameters were chosen to fit the spike properties of regular spiking pyramidal neurons: g_Le_ = 0.05, g_Na_ = 100, g_K_ = 40, g_Ca_ = 0.9, g_AHP_ = 0.05, V_L_ = −65, V_Na_ = 55, V_K_ = −80, V_Ca_ = 120, τ_Ca_ = 100.

#### Inhibitory neurons

The membrane potential of the inhibitory neurons was indicated as follows
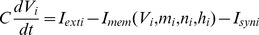
(14)where *V_i_* is membrane potential of an inhibitory neuron, and *m*, *h*, *n* are the gating variables for the ionic currents. The dynamics of the gating variables *m*, *h*, *n* were the same as for the excitatory neuron, the only differences between excitatory and inhibitory neurons were, first, that the inhibitory neurons lacked the *I_AHP_* current and, second, in the inhibitory interneurons membrane, time constants were shorter. Since the membrane time scale τ_mem_ is related to the membrane leak conductance g_L_ as τ_mem_∼1/g_L_, we have chosen in the inhibitory cells g_Li_ = 0.1 and in the excitatory cells g_Le_ = 0.05. Thus, the dynamics of the inhibitory cells was faster compared to the dynamics of the excitatory cells.

#### Synaptic currents

Synaptic currents to an excitatory neuron were then calculated according to
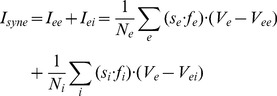
(15)Synaptic current to an inhibitory neuron:
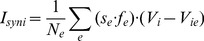
(16)where *V_e_* is membrane potential of an excitatory neuron, *V_i_* is membrane potential of an inhibitory neuron, *s_e_* is synaptic gating variables of AMPA currents, *s_i_* is synaptic gating variable of GABAa current, *f_e_*, *f_i_* are synaptic depression factors, and *V_ie_* = 80, *V_ee_* = 0, *V_ei_* = 0.

#### Synaptic models

Models for the AMPA receptor and GABA receptor were applied to simulate excitatory connections and inhibitory connections, respectively
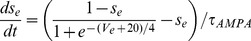
(17)

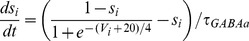
(18)where *s_e_* is a synaptic gating variable of AMPA current, and *s_i_* is a synaptic gating variable of GABAa current, *τ_AMPA_* = 2 and *τ_GABAa_* = 2.

#### V2 layer

The V2 layer contained 100 excitatory neurons. To model the excitatory neurons in V2, we used Hodgkin-Huxley equations with the same parameters as those used for layer one excitatory neurons.

#### Connections within layers

The excitatory and inhibitory neurons in V1 were connected through recurrent connections. Weights of the recurrent connections were distance-dependent and followed Gaussian distributions with s.d. = 10 neurons. The ratio between excitatory and inhibitory weights were chosen to balance recurrent excitation and inhibition in V1 neurons. The recurrent connections did not change with learning. There were no recurrent connections among V2 neurons.

#### Connections between layers

The excitatory neurons in V1 layer projected to the excitatory neurons in V2 layer through convergent feedforward connections. Probability of feedforward connections depended on corresponding locations of neurons in V1 and V2, and were randomly selected from Gaussian distributions with s.d. = 5 neurons, which resulted in a compact stimulus representation in V2 layer. The feedback connections from excitatory V2 neurons projected uniformly to all (excitatory and inhibitory) neurons in V1.

#### Learning

We simulated stimulus learning in the model by repeatedly presenting a stimulus with a particular orientation. Training the model strengthened excitatory feedforward connections from neurons representing the trained stimulus in V1 to neurons representing the trained stimulus in V2. Strong feedforward connections increased activity in V2 neurons, which in turn triggered plasticity of feedback connections.

#### Plasticity of feedforward connections

Feedforward connections from the excitatory V1 neurons to V2 neurons were adjusted according to a Hebbian learning rule. In the learning rule, we used intracellular calcium concentration as an indicator of a neural activity [48]

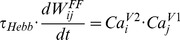
(19)where *τ_Hebb_* = 100 msec is time scale of Hebbian learning, 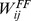 is the strength of a feedforward connection from an excitatory V1 neuron *j* to V2 neuron *i*, 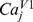 is intracellular calcium concentration in the excitatory V1 neuron *i*, 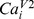 is intracellular calcium concentration in the excitatory V2 neuron *j*.

#### Plasticity of feedback connections

The feedback projections allowed V2 neurons to decrease activity in V1 when neurons in V2 were activated strongly by feedforward inputs from V1. In order to achieve this modulatory effect in the model, the strength of the feedback connections were adjusted according to a plasticity rule
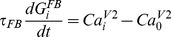
(20)where *τ_FB_* = 500 msec is a time scale of feedback plasticity, 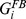 is the strength of the feedback connection from V2 neuron *i* to the excitatory and inhibitory V1 neurons, 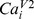 is an intracellular calcium concentration in the excitatory V2 neuron *i*, 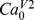 is a homeostatic value of the intracellular calcium concentration in the excitatory V2 neurons, which was calculated as a time averaged value of the intracellular calcium concentration in the excitatory V2 neurons.
